# Testing for the mediating role of endophenotypes using molecular genetic data in a twin study of ADHD traits

**DOI:** 10.1002/ajmg.b.32463

**Published:** 2016-05-27

**Authors:** Rebecca Pinto, Philip Asherson, Nicholas Ilott, Celeste H. M. Cheung, Jonna Kuntsi

**Affiliations:** ^1^ MRC Social, Genetic and Developmental Psychiatry Centre, Institute of Psychiatry, Psychology and NeuroscienceKing's College LondonLondonUK; ^2^ The Kennedy Institute of RheumatologyUniversity of OxfordOxfordUK; ^3^ Department of Psychology, Institute of Psychiatry, Psychology and NeuroscienceKing's College LondonLondonUK

**Keywords:** endophenotype, RTV, inhibition, inattention, hyperactivity–impulsivity, reading difficulties

## Abstract

Family and twin studies have identified endophenotypes that capture familial and genetic risk in attention‐deficit/hyperactivity disorder (ADHD), but it remains unclear if they lie on the causal pathway. Here, we illustrate a stepwise approach to identifying intermediate phenotypes. First, we use previous quantitative genetic findings to delineate the expected pattern of genetically correlated phenotypes. Second, we identify overlapping genetic associations with ADHD‐related quantitative traits. Finally, we test for the mediating role of associated endophenotypes. We applied this approach to a sample of 1,312 twins aged 7–10. Based on previous twin model‐fitting analyses, we selected hyperactivity–impulsivity, inattention, reading difficulties (RD), reaction time variability (RTV) and commission errors (CE), and tested for association with selected ADHD risk alleles. For nominally significant associations with both a symptom and a cognitive variable, matching the expected pattern based on previous genetic correlations, we performed mediation analysis to distinguish pleiotropic from mediating effects. The strongest association was observed for the rs7984966 SNP in the serotonin receptor gene (HTR2A), and RTV (*P* = 0.007; unadjusted for multiple testing). Mediation analysis suggested that CE (38%) and RTV (44%) substantially mediated the association between inattention and the T‐allele of SNP rs3785157 in the norepinephrine transporter gene (SLC6A2) and the T‐allele of SNP rs7984966 in HTR2A, respectively. The SNPs tag risk‐haplotypes but are not thought to be functionally significant. While these exploratory findings are preliminary, requiring replication, this study demonstrates the value of this approach that can be adapted to the investigation of multiple genetic markers and polygenic risk scores. © 2016 The Authors. *American Journal of Medical Genetics Part B: Neuropsychiatric Genetics* Published by Wiley Periodicals, Inc.

## INTRODUCTION

Attention‐deficit/hyperactivity disorder (ADHD) is characterized by developmentally inappropriate levels of inattention and hyperactivity–impulsivity. In addition to the core behavioral symptoms, ADHD is associated with multiple cognitive impairments [Kuntsi et al., 2010; Frazier‐Wood et al., 2012]. Family and twin studies have been largely successful in identifying cognitive phenotypes that capture the familial‐genetic risk in ADHD. Using a large sample of ADHD and control sibling pairs, we previously identified the separation of slow and highly variable reaction times (RTs) (reflecting lapses in attention) from commission and omission errors on a go/no‐go task (reflecting difficulties with response inhibition and failures to respond to a signal), into two familial factors [Kuntsi et al., 2010]. We also showed that the familial influences that ADHD shares with these cognitive impairment measures are largely separate from those that ADHD shares with IQ [Wood et al., 2010b]. More recently, we found that ADHD shares familial risks with reading difficulties (RD), and that this is largely independent of the familial risk factors shared between ADHD and IQ [Cheung et al., 2012]. Comparable findings were found in separate analyses on a large Dutch sample of ADHD and control sibling pairs using a different cognitive test battery. Two familial cognitive impairment factors again emerged, with the first capturing the speed and variability of responses across several tasks, and the second capturing aspects of executive functioning, such as working memory [Frazier‐Wood et al., 2012]. Familial influences on IQ were again largely separate: while the second familial factor captured 33% of the familial influences on IQ, 67% were not accounted for by the model [Frazier‐Wood et al., 2012].

Using a population‐based twin sample, we recently replicated the etiological separation of reaction time variability (RTV) and commission errors (CE) [Kuntsi et al., 2014] that we had previously observed in the sample with clinically diagnosed ADHD [Kuntsi et al., 2010]. Further analyses on the twin sample also replicated the finding of the genetic overlap between ADHD symptoms and cognitive impairments (including RD) being largely distinct from the effect of IQ [Paloyelis et al., 2010; Wood et al., 2010a]. Examining the etiological pathways separately for the two ADHD symptom dimensions, we found a strong genetic overlap between RTV and inattention symptoms [Kuntsi et al., 2014]. Although RTV was also significantly genetically associated with hyperactivity–impulsivity, the majority (55%) of the genetic covariance between inattention and RTV occurred independently of the genetic effects underlying hyperactivity–impulsivity [Kuntsi et al., 2014]. RD also shared a greater genetic risk with inattention versus hyperactivity–impulsivity symptoms [Paloyelis et al., 2010].

The comparability of these findings across both diagnostic and dimensional approaches to the ADHD phenotype is consistent with evidence from family and twin studies that the clinical diagnosis of ADHD reflects the extreme and impairing tail end of one or more continuous dimensions of psychopathology [Levy et al., 1997; Chen et al., 2008; Larsson et al., 2014]. Moreover, the findings highlight the importance of both shared and unique etiological pathways on the two ADHD symptom dimensions of inattention and hyperactivity–impulsivity [McLoughlin et al., 2011, 2007; Greven et al., 2011].

The cognitive phenotypes identified in these studies (such as RTV and CE) are often assumed to be intermediate phenotypes that lie on the causal pathway from genes to ADHD symptoms, since they are identified by the same genes that increase risk for ADHD. However, genetically correlated phenotypes may also reflect pleiotropy, whereby overlapping sets of genes give rise to multiple phenotypes that index genetic risk, but do not mediate between genes and the clinical phenotype [Kendler and Neale, 2010]. This distinction is important, as intermediate phenotypes, which are hypothesized to lie on the causal pathway from genes to a clinical phenotype, are potential targets for treatment and prevention of ADHD, whereas risk‐liability biomarkers reflect correlated phenotypes that play no direct causal role in the disorder [Kendler and Neale, 2010]. The distinction between pleiotropic and mediating genetic effects is widely assumed, but in the absence of formal testing it is not possible to distinguish between these effects [Asherson and Gurling, 2012].

As yet, only a few studies have investigated the mediating role of phenotypes in pathways from genetic variants to ADHD symptoms. One study found the association between the high‐activity catechol‐O‐methyltransferase gene (COMT) valine/valine genotype and antisocial behavior in ADHD was partially mediated by impaired social cognition, whereas impairments in executive control represented pleiotropic effects (i.e., genetically correlated but non‐mediating effects) [Langley et al., 2010]. Another study using a series of regression analyses on 11 different measures of executive functioning found no evidence that they mediated the association between three single nucleotide polymorphisms (SNPs) in the adrenergic receptor α−2A gene (ADRA2A) and ADHD affection status [Waldman et al., 2006]. In a study investigating ADHD symptoms, low conscientiousness and high neuroticism were found to partially mediate the association between a genetic composite (based on the number of risk alleles for DRD4, DAT1, and ADRA2A) and the inattentive symptoms of ADHD [Martel et al., 2010].

Overall, our knowledge of the causal links from genetic risk markers to ADHD remains very limited, in particular for the two ADHD symptom dimensions of inattention and hyperactivity–impulsivity considered separately. The aim of this study was, therefore, to address this gap by using genetic variants reported to be associated with ADHD in previous studies, to look for overlapping associations with both ADHD symptoms and cognitive performance. Then in a second step, we explicitly test for the mediating role of phenotypes in the association with ADHD genetic risk variants.

The selection of genetic variants for this study was based on candidate gene association findings with the dopaminergic, noradrenergic and serotoninergic neurotransmitter systems that had previously been reported to be associated with ADHD. Pharmacological, neuroimaging, and animal studies have established a role of these neurotransmitter systems in ADHD. For example, the main pharmacological action of stimulant mediation used to treat ADHD, such as methylphenidate and dexamphetamine, is to increase synaptic dopamine, generating much interest in genes involved in dopaminergic pathways. Adrenergic and serotonergic neurotransmission is also affected by ADHD medications. Moreover, there is evidence for a role of serotoninergic dysfunction in impulsive behavior in both human and animal studies [Koskinen et al., 2000a,2000b; Lesch and Merschdorf, 2000; Walderhaug et al., 2002]. Thus, converging evidence led to genes involved in the dopaminergic, noradrenergic and serotonergic systems to rank among the most frequently investigated in targeted candidate gene association studies of ADHD.

We selected SNPs and variable number tandem repeat (VNTR) polymorphisms that had previously provided evidence of association with ADHD in two or more studies (see Table I). The genetic variants were from genes involved in dopaminergic (dopamine transporter [SLC6A3], dopamine D4 receptor [DRD4], COMT, monoamine oxidase A [MAOA]), noradrenergic (norepinephrine transporter [SLC6A2]), and serotoninergic (serotonin 1B receptor [HTR1B], the serotonin 2A receptor [HTR2A], serotonin transporter [5‐HTT], tryptophan hydroxylase 2 [TPH2]) neurotransmission. We also selected SNPs in the synaptosomal‐associated protein 25 (SNAP‐25) gene, as it was one of the strongest associations that has emerged from meta‐analysis of candidate gene association studies [Gizer et al., 2009]. SNPs were also included from the Cadherin 13 gene (CDH13), which ranked among the top hits from genome‐wide association studies (GWAS) [Lasky‐Su et al., 2008b; Lesch et al., 2008] and is found under the significant linkage peak identified in a meta‐analysis of sibling‐pair studies [Zhou et al., 2008]. Finally, we tested for SNPs in the ciliary neurotrophic factor receptor (CNTFR), which had been reported to be associated in independent samples of children and adults with ADHD [Ribases et al., 2008]. Further analyses of SNPs across multiple genes and using polygenic risk scores is possible using genomewide association data, but at the time of completing the analyses presented here, further genotyping has not been completed.

**Table I:** Genetic Markers Chosen for Genotyping in Population‐Based Twin Sample^a^

Gene	Marker	Previous association with ADHD	Functional Status
CDH13	rs6565113	Association with total symptom count [Lasky‐Su et al., 2008b].	Intronic—no known function
CDH13	rs11646411	Associated with adult ADHD [Lesch et al., 2008].	Intronic—no known function
CNTFR	rs7036351	Associated with both adult and childhood ADHD [Ribases et al., 2008].	Intronic—no known function
DAT1	Intron 8 VNTR	Associated with clinical ADHD in meta‐analysis [Gizer et al., 2009].	Altered gene expression
DAT1	3′UTR VNTR	Associated with clinical ADHD in meta‐analyses [Yang et al., 2007; Gizer et al., 2009].	Altered gene expression
DRD4	Exon 3 VNTR	Associated with clinical ADHD in meta‐analyses [Li et al., 2006; Gizer et al., 2009]	Altered receptor response
HTR1B	rs6296	Associated with clinical ADHD in meta‐analysis [Gizer et al., 2009].	C>G synonymous Val287Val
MAOA	rs6323	Gene associated with ADHD [Domschke et al., 2005; Xu et al., 2007].	T‐allele associated with low activity MAOA
SLC6A2	rs3785143	Associated with clinical ADHD [Brookes et al., 2006; Kim et al., 2008; Xu et al., 2008].	Intronic—no known function
SLC6A2	rs3785157	Associated with clinical ADHD [Bobb et al., 2005; Xu et al., 2005], but with opposing alleles.	Intronic—no known function
SNAP‐25	rs1051312	Association found when five independent studies pooled together [Kim et al., 2007].	3′‐UTR: Altered gene expression
SNAP‐25	rs6077690	Association found when five independent studies pooled together [Kim et al., 2007].	Promoter region—functional significance unknown
SNAP‐25	rs1843809	Associated with clinical ADHD [Sheehan et al., 2005; Brookes et al., 2006], but with opposing allele.	Intronic—no known function
5HT2A	rs7322347	Associated with ADHD‐C subtype in children (not adults) [Ribases et al., 2009].	Intronic—no known function
5HT2A	rs7984966	Associated with ADHD‐C subtype in adults (not children) [Ribases et al., 2009].	Intronic—no known function

^a^ Excluded markers are not shown.

In this study, we illustrate a stepwise approach to identifying causal pathways from genetic markers to ADHD‐related ratings, by testing for associations with ADHD‐related quantitative traits and conducting selected mediation analyses in a general population twin sample. In the first step, informed by a series of quantitative genetic model‐fitting analyses in the same twin sample studying the etiological relationships across ADHD symptom subscales and cognitive measures [Paloyelis et al., 2010; Wood et al., 2010a; Cheung et al., 2014; Kuntsi et al., 2014], we selected ratings of inattention, hyperactivity–impulsivity, RD, RTV and CE to take forward to test for association with selected genetic risk variants. The selected risk variants were all previously reported to be associated with ADHD in two or more studies (Table I). In the second step, we conducted mediation analysis where genetic variants showed nominal overlapping associations with both a symptom domain and a cognitive variable that matched findings from the earlier genetic model‐fitting studies. The mediation analysis allows the distinction between a correlated risk‐liability indicator and intermediate phenotypes to be explicitly tested. We hypothesized that the same pattern of associations with clinical and cognitive phenotypes should emerge for specific markers, as those identified from genetic‐model fitting. Therefore, this study presents a proof of principle of the approach to combine quantitative and molecular genetic investigations to delineate causal pathways underlying ADHD‐related symptom ratings.

## MATERIALS AND METHODS

### Participants

Participants were from the Study of Activity and Impulsivity Levels in children (SAIL). Sampling methods and data collection procedures are described in detail elsewhere [Kuntsi et al., 2006]. The parents of all participating children provided informed consent, with ethical approval obtained from the Research Ethics Committee of the Institute of Psychiatry, King's College London, UK. The present analyses focused on a total of 1,312 children: 513 identical (monozygotic, MZ) twins (data for 255 complete twin pairs), 374 same‐sex non‐identical (dizygotic, DZ) twins (data for 184 complete twin pairs), 427 opposite‐sex DZ twins (207 complete twin pairs) and 22 singletons. Twin zygosity was determined using a parental‐report questionnaire with 95% accuracy, later verified using DNA [Price et al., 2000]. To check the error rate for twin zygosity status, we obtained the most recent data available from the overall TEDS twin dataset. Out of 3,728 twin pairs with genotype data, three twin pairs were erroneously classified as DZs when according to genotype data they were MZs, and no twin pairs were misclassified as MZs, giving an error rate of 0.08%. The mean age of participating children was 8.83 years (SD = 0.67), with a similar proportion of boys (49.5%) and girls. Children's IQs ranged from 70 to 158 (mean = 109.34, SD = 14.72).

### Measures

#### ADHD rating scales

Parents and teachers were asked to complete the Long Versions of Conners' Parent (CPRS‐R:L) and Teacher (CTRS:R:L) Rating Scales [Conners et al., 1998a,1998b]. From both scales, we used the nine‐item inattention and nine‐item hyperactivity–impulsivity DSM‐IV ADHD symptom subscales.

The vocabulary, similarities, picture completion and block design subtests from the WISC‐III were used to obtain an estimate of the child's IQ (prorated following procedures described by Sattler [1992]).

#### Reading difficulties

Reading Difficulties Questionnaire (RDQ) is a subscale of the Colorado Learning Difficulties Questionnaire [Willcutt et al., 2011]. This six‐item parent rating scale is part of an instrument screening for learning disorders. On a scale that ranges from 1 (never/not at all) to 5 (always/a great deal), parents reported the extent of their child's difficulties with spelling, learning letter names, sounding words out, and to what extent their child reads slowly, below expectancy level or has required extra help at school.

On each trial, one of two possible stimuli appeared for 300 ms in the middle of the computer screen. The child was instructed to respond only to the “go” stimuli and to react as quickly as possible, but to maintain a high level of accuracy. The proportion of “go” stimuli to “no‐go” stimuli was 4:1. The participants performed the task under three conditions (slow, fast, and incentive), matched for length of time on task. Herein, we present data from the slow condition, which had an inter‐stimulus interval of 8 sec and consisting of 72 trials, and the fast condition, with an inter‐stimulus interval of 1 sec and consisting of 462 trials. The order of presentation of the slow and fast conditions varied randomly across participants. We focus here on two variables obtained from the task: CE and RTV.

The baseline condition, with a foreperiod of 8 sec and consisting of 72 trials, followed a standard warned four‐choice RT task. A warning signal (four empty circles, arranged side by side) first appeared on the screen. At the end of the foreperiod (presentation interval for the warning signal), the circle designated as the target signal for that trial was filled (colored) in. The participant was asked to make a compatible choice by pressing the response key that directly corresponded in position to the location of the target stimulus. Following a response, the stimuli disappeared from the screen and a fixed inter‐trial interval of 2.5 sec followed. Speed and accuracy were emphasized equally. If the child did not respond within 10 sec, the trial terminated. A comparison condition with a fast event rate (1 sec) and incentives followed the baseline condition [Andreou et al., 2007]. Herein, we focus on RTV, obtained from the baseline condition.

### Selection of Clinical Variables for Tests of Genetic Associations

Tests of allelic association were performed on the exact same final variables used in corresponding quantitative genetic analysis in the same twin sample [Paloyelis et al., 2010; Cheung et al., 2014; Kuntsi et al., 2014]. In brief, parent and teacher ratings on corresponding ADHD subscales of the CPRS‐R:L and CTRS‐R:L [Conners et al., 1998a,1998b] were summed to obtain composite subscale measures that were more stable, reliable and situationally pervasive measures of ADHD behaviors. A total reading score was obtained from summing parent response to the six items of the RDQ. An overall RTV composite score was obtained across the two cognitive tasks by summing unstandardized RTV across the baseline conditions of the go/no‐go and fast task. An overall CE score was obtained across the two conditions by summing the percentage of CE across slow and fast conditions of the go/no‐go task. The final set of variables included in the molecular genetic analysis was inattention symptom scores, hyperactivity–impulsivity symptom scores, RD scores, RTV and CE. The measures were regressed to correct for the effects of age and sex (a standard twin modeling procedure) and non ADHD‐subscale ratings were further regressed for IQ. All measures were then transformed (with the exception of CE) using the optimized minimal skew “lnskew0” command in STATA.

### Genotyping

Nineteen polymorphisms were selected on the basis of previous reports of association with ADHD [Bobb et al., 2005; Domschke et al., 2005; Sheehan et al., 2005; Xu et al., 2008, 2007, 2005; Brookes et al., 2006; Li et al., 2006; Kim et al., 2007, 2008; Yang et al., 2007; Lasky‐Su et al., 2008a,2008b; Lesch et al., 2008; Ribases et al., 2008, 2009; Gizer et al., 2009]. SNPs associated with ADHD in these studies were included from the following genes: cadherin 13 (CDH13), ciliary neurotrophic factor receptor (CNTFR), DRD4, HTR1B, serotonin receptor (HTR2A), monoamine oxidase A (MAOA), norepinephrine transporter (SLC6A2), SLC6A3, SNAP‐25, and tryptophan hydroxylase 2 (TPH2). Variable Number Tandem Repeat (VNTR) polymorphisms nominated included: COMT Val158Met, DRD4 exon 3, SLC6A3 3′UTR, SLC6A3 intron 8, and 5‐HTTLPR.

DNA was extracted from buccal swabs (as described elsewhere [Freeman et al., 2003]). SNPs were genotyped using the Sequenom MassARRAY system. VNTR polymorphisms were genotyped manually using standard PCR based methods and resolved using agarose gel electrophoresis, as previously described [Xu et al., 2005; Brookes et al., 2006; Asherson et al., 2007].

Genotype error rates were estimated from genotype discordance rates within MZ twin pairs using PEDSTATS, a feature of the Quantitative Transmission Disequilibrium Test (QTDT) program [Abecasis et al., 2000]. SLC6A3 SNPs rs40184 and rs2625211 were excluded due to a high rate of MZ discordance (error rates of 5.70% and 4.18%, respectively). For the remaining 17 markers, the average MZ discordance error rate was 1.72%, (ranging from no error rates to 3.42%) and MZ concordance errors were re‐coded as missing genotypes. After these quality control steps, the 5‐HTTLPR VNTR was omitted as it had a high level (10.4%) of missing data. The missing rate for all other markers was 2.8–6.1%. All 16 markers used in the final analysis conformed to Hardy–Weinberg equilibrium (*P *> 0.01).

### Statistical Analyses

#### Genetic association

Tests of allelic association were performed using the QTDT program [Abecasis et al., 2000]. QTDT tests for association with quantitative phenotypes in a variance components framework. Three models of association were tested using a likelihood ratio test implemented in QTDT: the “Total Association” test (AT), the “Within‐Test” of association (AW), and the test of “Population Stratification” (AP). Overall association was tested using the AT model which assesses both the within‐pair differences as well as between pair sums (i.e., the correlation between phenotypic and genotypic differences and sums for each twin pair) and is the most powerful test in the absence of stratification effects. In contrast, the AW assesses the within component only. The within‐pair design of the AW means that it is unaffected by between‐family stratification effects, yet is less powerful than the AT in the absence of stratification. Stratification is tested using the AP test, which tests for a significant difference of the between pair component versus the within pair component of association. Stratification effects are dismissed when these components are equal (*P *> 0.05) and results are interpreted from the AT. Conversely, results are interpreted from the AW if significant stratification effects are detected [Mill et al., 2005].

To correct for multiple testing, a Bonferroni correction for the 16 independent markers analyzed was applied, requiring *P* < 0.003 to attain study‐wise statistical significance. We did not correct for the number of phenotypes tested as the majority of the quantitative traits are significantly correlated (0.12–0.59, *P* < 0.01). Furthermore, we set out to establish whether there was any evidence that the pattern of findings from the molecular genetic analysis would match that predicted by the pattern of genetic correlations in twin studies.

VNTR markers were tested using the “multi‐allelic” function in QTDT. This provides a single *P*‐value for tests of alleles with an allele frequency > 0.05. UNPHASED (http://www.mrc-bsu.cam.ac.uk/personal/frank/software/unphased/) [Dudbridge, 2008] was used to test X‐linked MAOA marker (rs6323) because QTDT cannot deal with such data. UNPHASED has no means for handling MZ twin data; therefore mean phenotypic scores for MZ pairs were used in these analyses, and MZ pairs were entered as singletons.

#### Mediation

**Figure 1:**
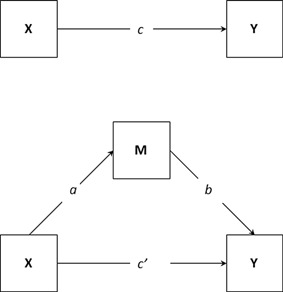
Path diagram for a mediation model. Note: adapted from [Fritz and MacKinnon, 2008]. X, independent variable; M, mediating variable; Y, dependent variable. The mediation model decomposes the total effect of X on Y (*c*) into two parts: the indirect effect of X on Y, reflected by *ab*, and the direct effect of X on Y with the effect of the mediator removed, reflected by *c*′.

Nominal genetic associations that overlapped across behavioral ratings and cognitive measures, and matched previous expectation from quantitative genetic findings were taken forward to test whether associations reflected pleiotropic or mediating genetic effects. Mediation analyses were conducted using a series of regression analyses performed in STATA. The mediation model is presented in Figure 1. The coefficient *c*′ represents the direct effect of X (the independent variable [predictor; here genotype]) on Y (the dependent variable; outcome), after controlling for the effect of M (the intervening variable; mediator). The coefficient *a* represents the effect of the SNP on the mediator, and the coefficient *b* represents the effect of the mediator on the outcome. Therefore, the mediated (or indirect) effect of the SNP on the outcome via the mediator is represented as *ab*. The total effect of the SNP on the outcome (including the indirect and direct effect) is represented by *c*, and is estimated as *ab* +* c*′.

Approaches to test mediation have been subjected to extensive research over the last two decades [Fairchild and MacKinnon, 2009]. In this paper, we calculate the indirect effect and test it for significance as suggested by Preacher and Hayes [2004], and adopt the product of coefficients approach to test the mediating effects of intervening variables on the genetic association of the number of risk alleles on questionnaire ratings, which tests the significance of the mediating variable by dividing the estimate of the indirect effect (*ab*) by its standard error, and comparing this value to a standard normal distribution to test for significance. There are multiple formulas to estimate the standard error (SE) of the mediated (*ab*) effect [see MacKinnon et al., 2002]. We adopt the most frequently used formula, derived by Sobel: √(*a* ^2^ × SE*b* ^2^) + (*b* ^2^ × SE*a* ^2^) (where SE*a* refers to the standard error of *a* and SE*b* refers to the standard error of *b*) [MacKinnon et al., 2002]. As stated above, *ab* is then divided by its standard error, and tested for significance using MacKinnon's z distribution. For these analyses, we adopted an alpha value of 0.05, for which a z value that does not lie between 1.96 and −1.96 is considered significant.

## RESULTS

### Association Analyses

Association findings are listed in Table II. The strongest association was found using the total test of association (AT) for the T‐allele of SNP rs7984966 in HTR2A with RTV (*P* = 0.007), although this did not withstand correction for multiple testing. The T‐allele of the same SNP in HTR2S was also nominally associated with RD (*P* = 0.02). The T‐allele of another HTR2A SNP (rs7322347) showed nominal association with inattention (*P* = 0.01). Increased RTV was also nominally associated with the G‐allele of SNP rs11646411 in CDH13 (*P* = 0.03). The T‐allele of one of the SNPs (rs3785143) in SLC6A2 showed a nominal AT association with CE (*P* = 0.03).

**Table II:** QTDT Association Analysis

		HYP‐IMP			INATT			RD			RTV			CE		
		AP	AT	AW	AP	AT	AW	AP	AT	AW	AP	AT	AW	AP	AT	AW
ADHD gene (markers)	df	*P* values			*P* values			*P* values			*P* values			*P* values		
CDH13 (rs6565113)	1	0.47	0.82	0.46	0.73	0.54	0.55	0.44	0.33	0.24	0.92	0.62	0.89	0.43	0.55	0.33
CDH13 (rs11646411)	1	0.82	0.39	0.87	0.58	0.85	0.66	0.08	0.80	0.14	0.17	**0.03**	0.73	0.65	0.77	0.60
CNTFR (rs7036351)	1	0.22	0.45	0.50	0.18	0.51	0.36	0.30	0.43	0.57	0.54	0.31	0.95	0.46	0.62	0.38
COMT VNTR	1	0.60	0.57	0.44	0.71	0.16	0.47	0.95	0.19	0.51	0.17	0.57	0.58	0.52	0.26	0.74
SLC6A3_3 VNTR	2	0.15	0.76	0.64	0.69	0.29	0.93	0.36	0.24	0.10	0.06	0.75	0.41	0.56	0.34	0.39
SLC6A3_8 VNTR	2	**0.04**	0.76	0.09	0.45	0.66	0.83	0.79	0.66	0.32	0.59	0.40	0.25	0.32	0.79	0.49
DRD4 VNTR	3	0.84	0.76	0.99	0.17	0.83	0.22	0.52	0.83	0.80	0.80	0.28	0.41	0.17	0.93	0.41
HTR1B (rs6296)	1	0.95	0.81	0.87	0.64	0.80	0.59	0.15	0.74	0.27	0.81	0.48	0.92	**0.05**	0.23	**0.02**
MAOA (rs6323)	NT	NT	NT	0.24	NT	NT	0.22	NT	NT	0.84	NT	NT	0.11	NT	NT	0.31
SLC6A2 (rs3785143)	1	0.09	0.66	0.09	0.36	0.80	0.48	0.10	0.36	0.32	0.23	0.12	0.93	0.41	**0.03**	0.10
SLC6A2 (rs3785157)	1	**0.03**	0.71	**0.04**	0.07	0.88	0.09	0.76	0.47	0.92	0.96	0.53	0.74	**0.03**	0.95	**0.05**
SNAP25 (rs1051312)	1	**0.03**	0.23	0.24	0.15	0.50	0.32	0.52	0.18	0.78	0.93	0.18	0.48	0.93	**0.08**	0.39
SNAP25 (rs6077690)	1	0.29	0.58	0.54	0.40	0.69	0.35	0.76	0.98	0.78	0.79	0.93	0.85	0.31	0.88	0.33
TPH2 (rs1843809)	1	0.14	0.96	0.22	**0.03**	**0.05**	0.25	0.19	0.17	0.07	0.77	0.31	0.83	0.54	0.90	0.55
HTR2A (rs7322347)	1	0.10	0.38	0.29	0.45	**0.01**	0.69	0.60	0.15	0.84	0.41	0.14	0.17	0.93	0.58	0.88
HTR2A (rs7984966)	1	0.11	0.75	0.12	0.11	0.09	0.40	0.82	**0.02**	0.37	0.65	**0.007**	0.43	0.72	0.81	0.82

*P* values < 0.05 are in bold.

df, difference in degrees of freedom between the null and alternative models.

HYP‐IMP, hyperactivity/impulsivity; INATT, inattention; RD, reading difficulties; RTV, reaction time variability; CE, commission errors; AP, test for population stratification; AT, total test of association; AW, within‐test of association; NT, not tested.

Taking into account the presence of population stratification effects, and therefore using the AW results, nominal AW associations were found for the T‐allele of the other SLC6A2 SNP (rs3785157) and both hyperactivity–impulsivity (*P* = 0.04) and CE (*P* = 0.05). In addition, nominal associations emerged between the G‐allele of SNP rs6296 in the HTR1B gene and increased CE (*P* = 0.02). Although we found an AT nominal association between the TPH2 SNP rs1843809 and inattention, since there was also evidence for significant population stratification, the AT association was ignored in favor of the AW estimate, which was non‐significant.

None of the associations withstood correction for the number of SNPs examined, requiring *P* < 0.003. However, both SNPs in SLC6A2 are of potential interest because of the overlapping nominal associations: the T‐allele in rs3785143 and CE (*P* = 0.03), and the T‐allele in rs3785157 and hyperactivity–impulsivity (*P* = 0.04) and CE (*P* = 0.05). In addition further, though weaker, trends emerged with hyperactivity–impulsivity (T‐allele in rs3785143; *P* = 0.09) and inattention (T‐allele in rs3785157; *P* = 0.09). In addition, HTR2A is of potential interest because of overlapping associations of the T‐allele in rs7984966 SNP with RTV (*P* = 0.007) and RD (*P* = 0.02), with a further trend for association with inattention (*P* = 0.09). Although all the overlapping associations are nominal and non‐significant when adjusted for multiple testing, the observed pattern of findings is consistent with previous findings derived from our previous genetic model‐fitting in the same sample. Therefore, we tested meditation models for these overlapping associations with both SLC6A2 SNPs and the HTR2A SNP rs7984966.

### Testing Candidate Mediating Pathways

**Figure 2:**
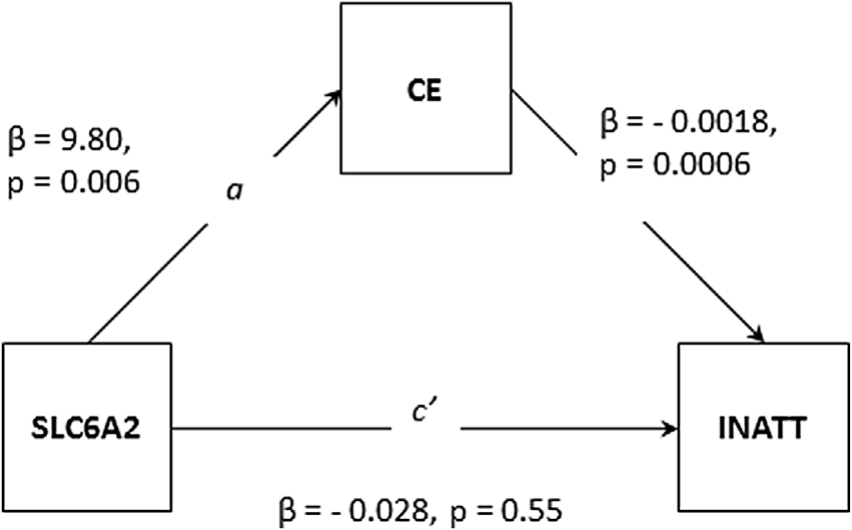
Mediation model of SLC6A2 on inattention via CE. Note: CE, commission errors; INATT, inattention.

**Figure 3:**
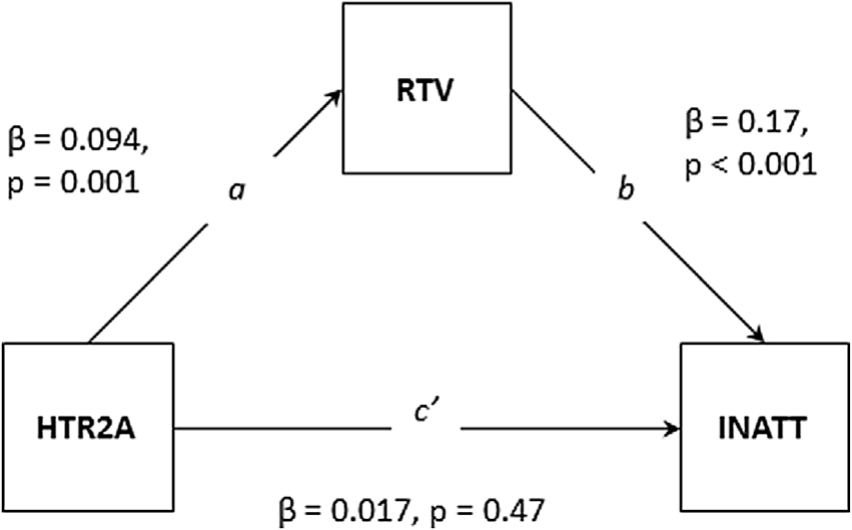
Mediation model of HTR2A on inattention via RTV. Note: INATT, inattention; RTV, reaction time variability.

QTDT associations were observed for the SLC6A2 SNP (rs3785157) with CE (*P* = 0.05) and both hyperactivity–impulsivity (*P* = 0.04), and to a lesser extent inattention (*P* = 0.09). Therefore, we tested two candidate pathways using two separate models with hyperactivity–impulsivity/inattention modeled as the outcome in alternative models. As the QTDT associations with rs3785157 were found using the AW test, within‐pair differences for phenotype and genotype (specifically risk alleles) were used in the regression tests for mediation. Although we found overlapping associations with the other SLC6A2 SNP (rs3785143) and both hyperactivity–impulsivity and CE, these could not be tested in a mediation model, as associations in QTDT were mixed (associations with CE obtained using AT test, and association with hyperactivity–impulsivity obtained using AW test). Associations were also observed for the HTR2A SNP rs7984996 with RTV (*P* = 0.007), RD (*P* = 0.02) and, to a lesser extent, inattention (0.09), and as these associations were found in the AT test, difference scores were not used for the regression analysis. Instead, individual genotype and phenotype data were used, and the “cluster” command employed to account for the genetic relationship within twin pairs. Only models where significant mediation is observed are presented (Figs. 2 and 3).

#### Testing CE as mediating the association between SLC6A2 and hyperactivity–impulsivity

In the first step of the regression analysis, we tested pathway *c* (see Fig. 1), and found that the SLC6A2 SNP rs3785157 predicted hyperactivity–impulsivity (pathway *c*: β = −0.0079, standard error (SE) = 0.044, *P* = 0.073). In the second regression step, we found that the SLC6A2 SNP rs3785157 significantly predicted CE (pathway *a*: β = 9.80, standard error (SE) = 3.53, *P* = 0.006). In the next step of the regression analysis, a hierarchical regression analysis was performed to test pathway *b* (β = −0.001, SE = 0.006, *P* = 0.079) *and c*′ (β = −0.07, SE = 0.045, *P* = 0.12). The indirect effect (*ab*) is estimated at −0.0097, and the SE of *ab* (SE*ab*) is estimated at 0.0065 (√(*b* ^2^ × SE*a* ^2^) + (*a* ^2^ × SE*b* ^2^)). The z statistic was estimated at −1.48 (*ab*/SE*ab*), *P* = 0.14. Therefore, the indirect (mediated) effect of CE on the association between SLC6A2 and hyperactivity–impulsivity was not significant.

#### Testing CE as mediating the association between SLC6A2 and inattention

The SLC6A2 SNP rs3785157 predicted inattention (β = −0.046, SE = 0.047, *P* = 0.33). The second step in this mediation model is a repeat of the second step in the previous model (as the SNP and mediator are the same), and so indicated that the SNP is significantly related to the mediator (CE) (*P* = 0.006). In the hierarchical regression analysis, CE significantly predicted inattention (β = −0.0018, SE = 0.0006, *P* = 0.003) and when controlling for the effects of CE on inattention, the effect of rs3785157 on inattention was β = −0.028, SE = 0.047, *P* = 0.55). The indirect effect (*ab*) is estimated at −0.017, and the SE of *ab* is estimated at 0.0086. The z statistic was estimated at −2.03, *P* = 0.04. Therefore, the indirect (mediated) effect of CE on the association between SLC6A2 and inattention was significant (Fig. 2). The proportion of the total effect between the SLC6A2 SNP rs3785157 and inattention that is mediated by CE is 38% ((ab/c) × 100).

#### Testing RTV as mediating the association between HTR2A and inattention

Regression analyses revealed that the HTR2A SNP rs7984966 predicted inattention (pathway *c*: β = 0.037, SE = 0.024, *P* = 0.13), and RTV (pathway *a*: β = 0.094, SE = 0.028, *P* = 0.001). In the hierarchical regression analysis, RTV significantly predicted inattention (β = 0.17, SE = 0.024, *P* < 0.001) and when controlling for the effects of RTV on inattention, the effect of rs7984966 on inattention was β = 0.017, SE = 0.024, *P* = 0.47). The mediated effect of RTV on the association between the HTR2A SNP rs7984966 and inattention was significant *(ab* = 0.016, z = 2.99, *P* = 0.003) (Fig. 3). The proportion of the effect of rs7984966 on inattention accounted for by RTV was 44%.

#### Testing inattention as mediating the association between HTR2A and RD

In the first step of the regression analysis, we tested pathway *c*, and found that the HTR2A SNP rs7984966 predicted RD (β = 0.11, standard error (SE) = 0.05, *P* = 0.03). In the second regression step, we found that the SNP predicted inattention (pathway *a*: β = 0.037, standard error (SE) = 0.024, *P* = 0.13). In the next step of the regression analysis, a hierarchical regression analysis was performed to test pathway *b* and *c′*. Inattention significantly predicted RD (β = 0.98, SE = 0.06, *P* < 0.001), and when controlling for the effects of inattention on RD, the effect of the HTR2A SNP rs7984966 on RD was β = 0.079, SE = 0.045, *P* = 0.08. The mediated effect of inattention on the association between the HTR2A SNP rs7984966 and RD was not significant *(ab* = 0.036, z = 1.51, *P* = 0.13).

## DISCUSSION

Informed by our previous genetic model‐fitting results [Paloyelis et al., 2010; Cheung et al., 2014; Kuntsi et al., 2014], we investigated the molecular genetic correlates of the two ADHD symptom domains of inattention and hyperactivity–impulsivity with key cognitive impairments we have previously shown to be associated with the genetic risk for ADHD. Using genetic risk alleles from candidate genes previously reported to be associated with ADHD, we found that several of the genetic variants showed nominal associations across phenotypes previously shown to share genetic risk factors with ADHD. Although none of these withstood correction for multiple testing, the observed associations are of potential interest because the pattern of findings is consistent with previous findings derived from our genetic model‐fitting analyses in the same sample. For this reason, we conducted mediation analyses on selected SNPs where there was an overall pattern of findings in line with previous twin model‐fitting findings.

From the mediation tests we obtained preliminary evidence that RTV mediated the association between the T‐allele of rs7984966 in HTR2A and inattention, and CE mediated the association between the T‐allele of rs3785157 in SLC6A2 and inattention. In contrast, there was no evidence for the mediating role of inattention in the association between rs7984966 in HTR2A and RD, or for CE in the association between rs3785158 in SLC6A2 and hyperactivity–impulsivity, suggesting pleiotropic (non‐mediating) genetic effects.

The strongest association was between the T‐allele of SNP rs7984966 in HTR2A and RTV, which was also associated with RD, and at trend level with inattention. A further SNP in HTR2A (rs7322347) was also associated with inattention. These SNPs have been associated both in single marker and haplotype (multiple markers) analyses with ADHD (DSM‐IV combined type) in both children and adults [Ribases et al., 2009]. However, our associations were with the opposite alleles to those reported in the previous literature [Ribases et al., 2009]. It is interesting to note that this finding in the opposite direction to the expected effect may mirror DRD4 findings in the literature, which suggest that the absence of the ADHD risk allele is associated with superior RTV performance [Bellgrove et al., 2008; Kebir et al., 2009; Kebir and Joober, 2011].

Overlapping nominal associations were also found for two SNPs in SLC6A2: both SNPs were associated with CE but not associated with either RTV or RD, and one SNP (rs3785157) was also further associated with both ADHD behavioral dimensions (although the association with inattention was at a trend level). In the analysis, we identified the T allele of the SLC6A2 SNP, rs3785157, as the risk allele, in line with some studies [Bobb et al., 2005], but opposing others [Xu et al., 2005]. The association of both SLC6A2 SNPs with CE are in line with a recent study of Korean children with ADHD, which found an association with SLC6A2 and CE [Song et al., 2011].

Overall, the pattern of findings we observed (overlapping associations with RTV, inattention and RD; overlapping associations with CE and both inattention and hyperactivity–impulsivity; and non‐overlapping associations with CE and either RTV or RD) is in line with the results from our quantitative genetic analyses [Paloyelis et al., 2010; Cheung et al., 2014; Kuntsi et al., 2014].

While these findings are preliminary, based on weak nominal significance with individual genetic variants, the similar pattern of findings between the molecular and quantitative genetic findings is of interest and met our criteria for conducting mediation tests. We therefore performed mediation analyses to formally distinguish if overlapping associations reflect pleiotropic or mediating effects [Kendler and Neale, 2010; Langley et al., 2010] for genetic markers showing overlapping nominal associations (including trend level findings consistent with the findings from twin model fitting) with both a symptom domain and a cognitive variable.

Although overlapping associations suggested that rs3785157 in SLC6A2 may be a potential genetic candidate contributing to the association of CE with both ADHD behavioral dimensions, our further analyses suggested that the overlapping nominal genetic associations with hyperactivity–impulsivity reflect pleiotropic genetic effects. In contrast, CE mediated 38% of the effect of SLC6A2 on inattention. Another overlapping set of associations was observed for rs7984966 in HTR2A with RTV, RD and inattention, implicating it as a potential candidate marker, in line with our previous quantitative genetic finding that the association of RTV and RD with ADHD largely reflects genetic influences shared with inattention that are distinct from those underlying hyperactivity–impulsivity [Paloyelis et al., 2010; Cheung et al., 2014; Kuntsi et al., 2014]. Mediation analysis indicated that inattention did not significantly mediate the effects of HTR2A on RD. In contrast, RTV significantly mediated a substantial proportion (44%) of the effect of HTR2A on inattention symptoms. We therefore obtained preliminary evidence for CE and RTV as intermediate phenotypes on the pathway to inattention, respectively from the SLC6A2 and HTR2A genes.

In common with previous studies incorporating quantitative assessments of ADHD within molecular genetic investigations, power to detect genetic associations was limited due to a small sample size. Power for this sample was estimated using the genetic power calculator from Purcell et al. [2003] and found to be around 80% for nominal significance (*P *= 0.05) for genetic loci explaining around 0.5% of the phenotypic variance. The genetic associations, therefore, need to be treated with considerable caution, as none of the individual associations reported withstood correction for multiple testing, and did not account for the multiple phenotypes also targeted. Furthermore, some of the allelic specific associations we identified were not in the same direction as predicted by the previous literature. Despite these limitations, we found nominal associations with previously implicated ADHD susceptibility genes and psychometrically robust ADHD‐related phenotypes, selected on the basis of quantitative genetic model‐fitting analyses. Since the pattern of findings was consistent with those from the previous quantitative genetic analyses these are less likely to have arisen by chance.

It is essential that these findings are extended and replicated; candidate gene associations are notoriously fickle and this could potentially be the case with the present findings. The next stage of this approach is already underway with collaboration between international investigators generating large samples, with clinical phenotypic data (ADHD symptom counts), cognitive measures and genome‐wide association data. The findings presented here are nevertheless encouraging and outline a general strategy that can easily be adapted to the investigation of multiple genetic markers and polygenic risk scores. Matching genetic association findings to the predicted patterns of genetically correlated traits, from twin model fitting and other quantitative genetic designs, may help to delineate the role of specific gene or gene pathways, and provides a framework for testing for the causal (mediating) role of cognitive endophenotypes on ADHD.
